# Immersive and Nonimmersive Virtual Reality–Assisted Active Training in Chronic Musculoskeletal Pain: Systematic Review and Meta-Analysis

**DOI:** 10.2196/48787

**Published:** 2024-08-19

**Authors:** Hermione Hin Man Lo, Mengting Zhu, Zihui Zou, Cho Lee Wong, Suzanne Hoi Shan Lo, Vincent Chi-Ho Chung, Samuel Yeung-Shan Wong, Regina Wing Shan Sit

**Affiliations:** 1 Nethersole School of Nursing Faculty of Medicine The Chinese University of Hong Kong Hong Kong China (Hong Kong); 2 Jockey Club School of Public Health and Primary Care Faculty of Medicine The Chinese University of Hong Kong Hong Kong China (Hong Kong)

**Keywords:** virtual reality, VR, physical therapy, musculoskeletal, pain, physiotherapy, chronic pain, musculoskeletal pain, low back pain, neck pain, osteoarthritis, knee pain, shoulder pain, disability, kinesiophobia, arthritis, systematic, review methods, review methodology, immersive, simulation, simulations

## Abstract

**Background:**

Virtual reality (VR) in different immersive conditions has been increasingly used as a nonpharmacological method for managing chronic musculoskeletal pain.

**Objective:**

We aimed to assess the effectiveness of VR-assisted active training versus conventional exercise or physiotherapy in chronic musculoskeletal pain and to analyze the effects of immersive versus nonimmersive VR on pain outcomes.

**Methods:**

This systematic review of randomized control trials (RCTs) searched PubMed, Scopus, and Web of Science databases from inception to June 9, 2024. RCTs comparing adults with chronic musculoskeletal pain receiving VR-assisted training were included. The primary outcome was pain intensity; secondary outcomes included functional disability and kinesiophobia. Available data were pooled in a meta-analysis. Studies were graded using the Cochrane Risk-of-Bias Tool version 2.

**Results:**

In total, 28 RCTs including 1114 participants with some concerns for a high risk of bias were identified, and 25 RCTs were included in the meta-analysis. In low back pain, short-term outcomes measured post intervention showed that nonimmersive VR is effective in reducing pain (standardized mean difference [SMD] –1.79, 95% CI –2.72 to –0.87; *P*<.001), improving disability (SMD –0.44, 95% CI –0.72 to –0.16; *P*=.002), and kinesiophobia (SMD –2.94, 95% CI –5.20 to –0.68; *P*=.01). Intermediate-term outcomes measured at 6 months also showed that nonimmersive VR is effective in reducing pain (SMD –8.15, 95% CI –15.29 to –1.01; *P*=.03), and kinesiophobia (SMD –4.28, 95% CI –8.12 to –0.44; *P*=.03) compared to conventional active training. For neck pain, immersive VR reduced pain intensity (SMD –0.55, 95% CI –1.02 to –0.08; *P*=.02) but not disability and kinesiophobia in the short term. No statistical significances were detected for knee pain or other pain regions at all time points. In addition, 2 (8%) studies had a high risk of bias.

**Conclusions:**

Both nonimmersive and immersive VR–assisted active training is effective in reducing back and neck pain symptoms. Our study findings suggest that VR is effective in alleviating chronic musculoskeletal pain.

**Trial Registration:**

PROSPERO CRD42022302912; https://www.crd.york.ac.uk/prospero/display_record.php?RecordID=302912

## Introduction

Chronic musculoskeletal pain is a worldwide health problem with varying effects on physical, psychological, and social functioning [1,2]. According to the Global Burden of Disease Study of 2019, chronic musculoskeletal pain, especially low back pain, is the leading cause of disability worldwide, resulted in 149 million years of life lost [3]. The burden is expected to increase with an aging population and longevity [4].

Multimodal care is often needed in the management of chronic pain, with the aim of maintaining physical functioning and psychosocial well-being [5]. Exercise therapy is a well-known nonpharmacological modality in chronic pain management, with positive effects on pain intensity, physical function, sleep, and the quality of life [6]. Its additional benefits on happiness through the release of endorphins, serotonins, dopamine, and other “reward” chemicals have been demonstrated among individuals with chronic pain and depression [7,8]. Recent studies have suggested that technological advancements may increase the attractiveness of these active physical training programs, thus further improving compliance, adherence, and clinical outcomes [9,10].

Virtual reality (VR) is a digital technology that creates “a sense of presence in an computer-generated, three-dimensional, interactive environment with different immersive conditions” through head-mounted devices (HMDs), body-tracking sensors, and direct user input devices [11]. The use of VR promotes physical activity by increasing energy expenditure for fitness [12]. The capability of VR to reduce pain has mostly been attributed to active distraction (visual, auditory, and tactile input through interaction with a VR environment), which is understood to reduce resources available for the perception and elaboration of pain, thus diminishing subjective pain experience [13]. Gaming technology with motivational and affectively rewarding elements, as well as the goal-oriented interaction with a virtual environment, is suggested to have greater pain reduction [14].

An increasing number of trials have evaluated the role of VR-assisted active training in chronic musculoskeletal pain. Yet, within VR applications, an important distinction can be made between immersive and nonimmersive media, which differ in spatial presence [15]. With immersive technology, participants view the full panorama and are essentially inside the created environment. In a nonimmersive environment, virtual content is based on how the device (personal computer, smartphone, or tablet) is moved or rotated, and participants are only external observers. Whether immersive or nonimmersive VR is better for pain management remains unclear.

The aim of this systematic review was to assess the effectiveness of VR-assisted active training versus conventional active controls for musculoskeletal pain in different regions and to analyze the effects of immersive versus nonimmersive VR on validated pain outcomes.

## Methods

### Study Design

This systematic review followed PRISMA (Preferred Reporting Items for Systematic Reviews and Meta-Analyses) 2020 guidelines [16]. The protocol was registered in the International Prospective Register of Systematic Reviews (PROSPERO) registry (ID CRD42022302912).

### Search Strategy

Two independent reviewers (authors HHML and ZZ) independently screened papers from PubMed, Scopus, and the Web of Science. A systematic search was conducted from inception to June 9, 2024. A backward reference search was used to increase the yield of eligible studies. Only English papers with full text available were included.

Our search strategy had 2 components for “chronic primary musculoskeletal pain” and “virtual reality.” Keywords for the population were “cervical pain” OR “neck pain” OR “shoulder pain” OR “thoracic pain” OR “back pain” OR “low back pain” OR “joint pain” OR “arthralgia” OR “knee pain” OR “ankle pain” OR “limb pain” OR “osteoarthritis NOT structural” or “degenerative joint.” Keywords for the intervention were “virtual reality” OR “augmented reality” OR “mixed reality.” Please refer to Multimedia Appendix 1 for detailed search strategies.

### Eligibility Criteria

All parallel or cross-over randomized controlled trials (RCTs) that evaluated the effectiveness of VR-assisted active training in chronic musculoskeletal pain were included. Our review included both 2-arm and multiarm trials [17]. Chronic musculoskeletal pain was defined as pain that lasts for more than 3 months persistently or intermittently, including regional pain (joints, limbs, back, or neck), a degenerative joint condition (eg, osteoarthritis), and musculoskeletal complaints that fall under the “chronic primary pain” classification of the *International Classification of Disease, 11th Revision* [18]. We included all VR interventions that create synchronized motion-based interactions with computer-generated objects and provide a sense of “presence” for users. Presence is defined as VR users’ feeling of being immersed in a computer-generated environment via HMDs (immersive) or screens (nonimmersive), user input devices, body motion sensors, or commercial video game consoles [19,20]. To compare the effects of VR-assisted active training, we included studies with control groups using conventional exercise therapy or physiotherapy. Detailed inclusion and exclusion criteria are shown in Textbox 1.

**Inclusion and exclusion criteria.**

**Inclusion criteria**

- Randomized controlled trials (RCTs)
- Aged ≥18 years, with chronic musculoskeletal pain
- Virtual reality (VR)–assisted exercise therapy or physiotherapy
- Active training in comparison groups, including exercise therapy or conventional active physiotherapy

**Exclusion criteria**

- Cancer-related pain or autoimmune arthritis
- Psychotherapies
- Waitlist controls/daily life routine
- Passive physiotherapy in controls
- VR-assisted controls

### Outcome Measures

The primary outcome was pain intensity. To be eligible, studies had to measure pain intensity using the Visual Analog Scale, the Numerical Rating Scale, the McGill Pain Questionnaire, the Chronic Pain Grade Scale, or other validated questionnaires [21,22]. Secondary outcomes were functional disability measured using disease-specific scales, such as the Roland-Morris Disability Questionnaire (RMDQ) [23] or the Oswestry Disability Index (ODI) [24] for chronic low back pain; the Neck Disability Index or the Neck Pain Disability Scale for chronic neck pain [25-27]; the Western Ontario and McMaster Universities Osteoarthritis Index (WOMAC) for knee pain [28]; the Disabilities of the Arm, Shoulder, and Hand Questionnaire for shoulder pain [29]; and other validated questionnaires. Psychological status was measured through kinesiophobia, an emotional and cognitive factor that leads to maladaptive behaviors [30,31]; studies have shown that kinesiophobia is associated with more pain and disability and a lower quality of life [30]. Kinesiophobia was assessed using the Fear-Avoidance Beliefs Questionnaire [32] and the TAMPA Scale of Kinesiophobia [33,34].

### Study Selection and Data Extraction

Studies retrieved from the databases were uploaded to Covidence online systematic review software (Veritas Health Innovation). Two independent reviewers (HHML and ZZ) performed title and abstract screening of the retrieved literature for potentially eligible trials. The full text of selected papers was then retrieved and screened against the inclusion and exclusion criteria. Both reviewers (HHML and ZZ) independently judged the eligibility of the full text retrieved, and disagreements between the 2 reviewers were resolved by a third reviewer (author RWSS).

Data were then extracted from the selected papers by the first reviewer (HHML) and cross-checked by the second reviewer (ZZ). The extraction table headings included the first author, the year of publication, chronic pain subtypes, the sample size analyzed, the intervention group, the control group, the dosage of interventions, the mean age, outcomes (in terms of the mean difference [MD]), and assessment time points in weeks. Two independent reviewers (HHML and ZZ) then extracted MDs and SDs for the following domains: (1) pain intensity, (2) disability, and (3) kinesiophobia.

### Risk-of-Bias Assessment

Selected studies were assessed according to the intention to treat using the Cochrane Risk-of-Bias Tool version 2 (RoB 2). RoB 2 covers all types of biases that affect the results of RCTs: (1) bias due to randomization, (2) bias caused by deviations from intended interventions, (3) missing outcome data that results in bias, (4) bias in outcome measurement, and (5) the selection bias of reported results. Two reviewers (HHML and MZ) assessed the risk of bias independently for the selected papers, while a third reviewer (RWSS) acted as the arbitrator. A conclusion for risk-of-bias judgment was made by consensus.

Publication bias was assessed for the meta-analysis with 10 or more RCTs and was determined by constructing a funnel plot with the standard error against the effect size [35].

### Quality of Evidence

The Grading of Recommendation Assessment, Development, and Evaluation (GRADE) approach was used to assess the quality of evidence with GRADEpro software [36]. The quality of evidence for pain intensity, disability, and kinesiophobia at different time points was assessed separately. Evidence was downgraded if (1) the risk of bias was apparent (1 study showed a high risk, and 24 studies showed some concerns); (2) inconsistency was demonstrated, with *I*^2^>50%; (3) there was indirectness in participants or comparators (ie, whether participants or comparators aligned and compared with our research question, eg, participants had chronic musculoskeletal pain and comparators used conventional active training/physical therapy); (4) evidence of imprecision (when the effect size was large, ie standardized mean difference [SMD]>0.8 or MD>minimally clinically important difference [MCID; 95% CI], but the total sample size was small) [37]; or (5) there was publication bias (funnel plots were visually inspected when at least 10 trials were included in the meta-analysis). An overall GRADE rating was agreed upon (HHML and MZ) for each included study at 4 levels: very low, low, moderate, and high.

### Statistical Analysis

All meta-analyses were conducted using Review Manager (RevMan version 5.4) software (Cochrane). Pairwise meta-analysis was performed using a random effects model according to nonimmersive or immersive VR interventions [38]. Pain intensity, disability, and kinesiophobia were analyzed according to pain regions. Regarding the assessment time points, analysis was conducted in the immediate postintervention period (short term) and at 6 months (intermediate term). In our study, we defined short-term pain outcomes at 12 weeks and intermediate-term pain outcomes at 6 months. These time frames were established based on our clinical experience and the expected response timeline for the pain interventions used. We chose a 12-week period for assessing short-term pain outcomes as it aligns with the typical clinical trajectory observed in patients receiving pain interventions. During this period, patients are likely to experience the initial benefits of the treatment, and early therapeutic effects are most evident. The 24-week mark was selected to represent intermediate-term pain outcomes based on the continued progression of therapeutic effects and sustained pain relief observed in clinical practice. SMDs were used to measure continuous outcomes with more than 1 measuring scale. The SMD was clinically interpreted as Cohen d (SMD 0.2 was considered a small effect, 0.5 was considered a moderate effect and clinically important, and 0.8 was considered a large effect) [39,40]. Weighted mean differences (MDs) with 95% CIs were used to measure continuous outcomes within a domain on similar scales, and relevant minimal clinical important differences were used to assess clinical significance. Heterogeneity between studies was further explored by referring to I^2^ statistics for all outcomes: *I*^2^ <25%, low heterogeneity; *I*^2^=25%-50%, moderate heterogeneity; and I^2^>50%, high heterogeneity [41].

## Results

### Eligible Studies

**Figure 1:**
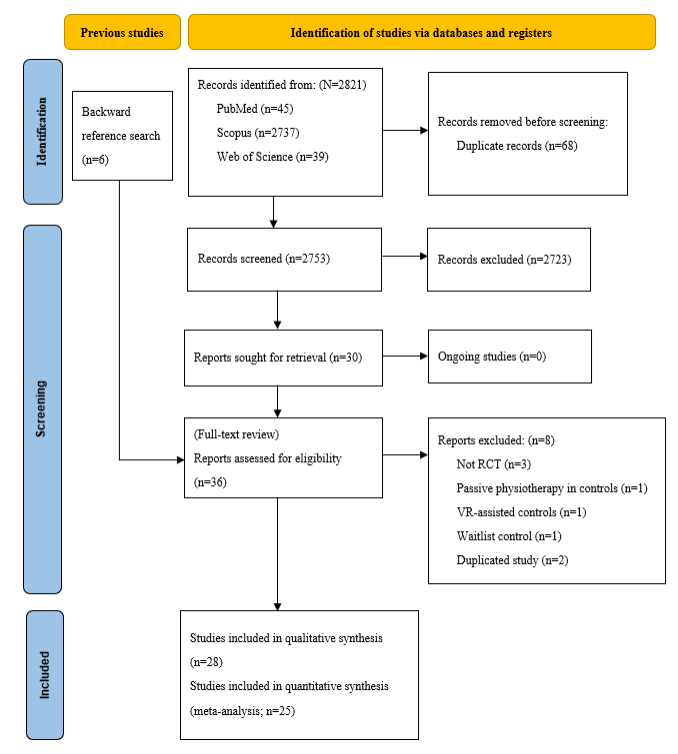
PRISMA flowchart for study selection. PRISMA: Preferred Reporting Items for Systematic Reviews and Meta-Analyses; RCT: randomized control trial; VR: virtual reality.

A total of 2753 papers were screened after removing duplicates. After title and abstract screening, 30 (1.1%) papers were selected for full-text retrieval and screening. The remaining reports and 6 papers from the backward reference search were screened by 2 independent reviewers (HHML and MZ), and 8 (22.2%) papers were excluded for the following reasons: not RCTs (n=3, 37.5%), passive physiotherapy in controls (n=1, 12.5%), VR-assisted controls (n=1, 12.5%), waitlist controls (n=1, 12.5%), and duplicate studies (n=2, 25%). A total of 28 (93.3%) of 30 studies were included in the final qualitative synthesis and 25 (83.3%) of the 30 studies were included in the meta-analysis synthesis (Figure 1) [42-65]. Furthermore, 3 (10.7%) studies could not be pooled: 1 (33.3%) single-out nonimmersive VR study on neck pain [66], 1 (33.3%) RCT on different joint pains [60], and 1 (33.3%) study on shoulder pain [59].

### Characteristics of Included Trials

Table S1 in Multimedia Appendix 2 summarizes all the 28 RCTs [42-69] selected. The mean age of the study sample was 42.50 (SD 3.49) years in the VR intervention group and 42.93 (SD 3.73) years in the control group. The sample sizes ranged from 19 (1.7%) to 84 (7.3%), with a total sample size of 1144. The average proportion of female participants was 49.3% (n=564) in all trials, excluding 3 (10.7%) trials [42,58,67] that did not provide participants’ sex ratio. In addition, 18 (64.3%) studies [42-47,54-63,66-68] used nonimmersive VR, while 8 studies [48-53,64,65,69] used immersive VR. Each VR session lasted from 10 to 45 minutes (mean 26.54, SD 9.05), except 4 (28.6%) studies [43,48,49,65] that did not report the VR intervention time. The mean total number of sessions was 15.46 (SD 7.18), and the frequency ranged from a single session to 5 sessions/week. Control groups included conventional physical therapy, such as balance and core stabilization training, walking, and audio-guided, isokinetic dynamometer–assisted, proprioceptive, kinematic, and sensorimotor exercises. Furthermore, 4 (14.3%) studies [53,60,64,69] reported no adverse events during the trials. Minor simulator sickness symptoms (eg, dizziness, nausea, headache) were reported in 2 (7.1%) studies [50,51].

### Risk-of-Bias Assessment

**Figure 2:**
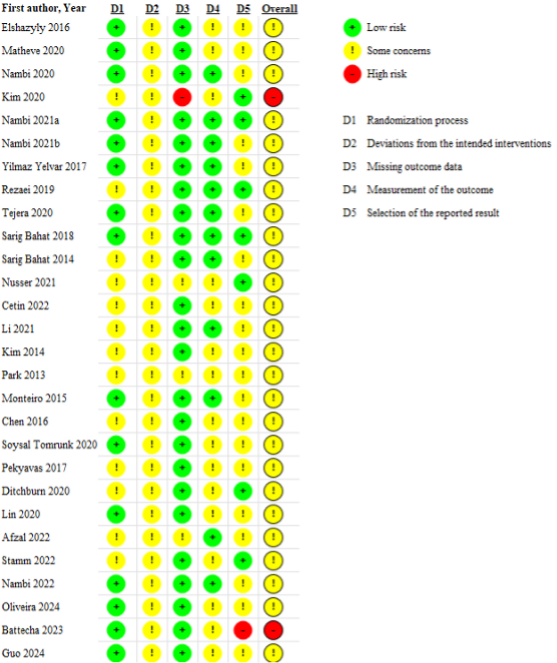
Risk-of-bias assessment of the included studies.

All included studies were assessed with RoB 2 (Figure 2). Overall, 26 (92.9%) studies [42-44,46-63,66-70] were rated as having “some concerns” of bias, and 2 (7.1%) studies [45,65] were rated as having a “high risk” of bias. In the domain of “bias arising from the randomization process,” 15 (53.6%) studies [42-44,46-49,51,57,61,63,65,67-69] had low bias and 13 (46.4%) studies [45,50,52-56,58-60,62,64,66] had some bias. In the domain of “deviations from the intended interventions,” all 28 (100%) studies had some bias due to failure to blind participants or delivery of interventions. In the domain of “missing outcome data,” 24 (85.7%) studies [42-44,46-51,53-55,57-61,63-66,68,69] had low risk of bias, 3 (10.7%) studies [52,56,62] had some concerns regarding bias, and 1 (3.6%) study [45] had high risk of bias. In the domain of “bias arising from measurement of the outcome,” 12 (42.9%) studies [44,46-51,53-55,57-61,63,64,66,67] had low bias and 16 (57.1%) studies [42,43,45,52,53,55,56,62,65,68,69] had some bias. The absence of blinding outcome assessors or data analysts was observed in studies with some bias. In the domain of “selection of reported results,” 7 (25%) studies [45-47,51,52,60,64,66] had low risk of bias, 20 (71.4%) studies [42-44,47,49,50,53-59,61-63,67-69] had some concerns regarding bias, and 1 (3.6%) study [65] had high risk of bias. This domain was mainly affected by the absence of prespecified analysis plans in preliminary and pilot studies.

### Pain Region: Back

#### Pain Intensity

**Figure 3:**
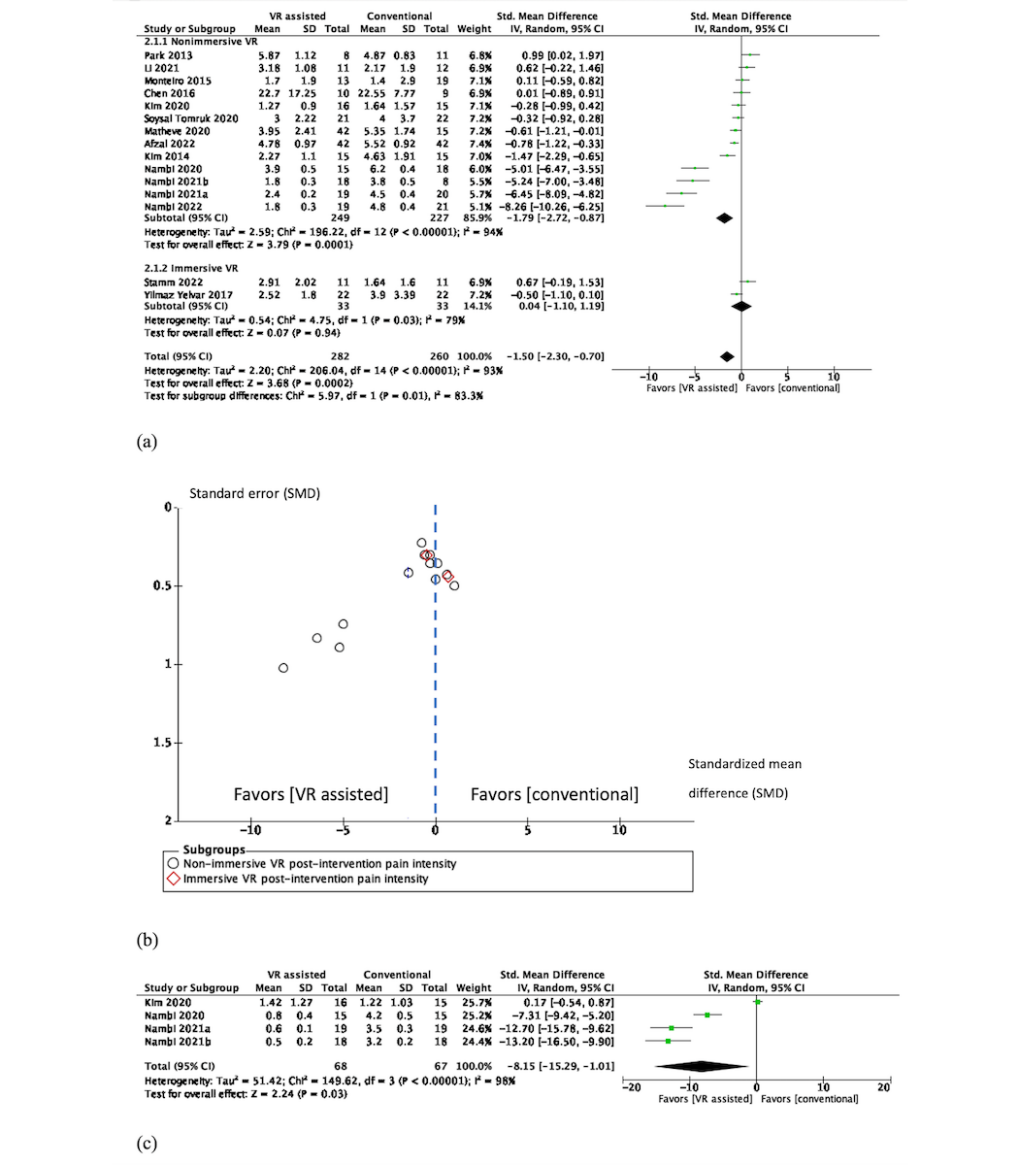
(a) Short-term effects of VR-assisted vs conventional active training in back pain intensity (nonimmersive VR via flat screen; immersive VR via HMDs). (b) Funnel plots for short-term effects of VR-assisted vs conventional active training in back pain intensity. (c) Intermediate effects of nonimmersive VR–assisted vs conventional active training in back pain intensity. HMD: head-mounted device; VR: virtual reality.

In the short term, 15 (53.6%; n=563, 49.2%, participants) of the 28 RCTs [43-48,54-58,62-64,67] were eligible for pooling. Results from 13 (46.4%) RCTs favored the use of nonimmersive VR in reducing pain intensity (SMD –1.79, 95% CI –2.72 to –0.87; *P*<.001), with high heterogeneity (*I*^2^= 94%) compared to active training. Results of immersive VR from 2 (7.1%) RCTs were statistically insignificant (SMD 0.04, 95% CI –1.10 to 1.19; *P*=.94), as shown in Figure 3a. Visual inspection of funnel plots (Figure 3b) indicated publication bias in our meta-analysis.

In the intermediate term, 4 (14.3%; n=135, 11.8%, participants) RCTs [44-47] were eligible for pooling; again, results favored nonimmersive VR in reducing pain intensity (SMD –8.15, 95% CI –15.29 to –1.01; *P*=.03), with high heterogeneity (*I*^2^= 98%), as shown in Figure 3c.

#### Functional Disability

**Figure 4:**
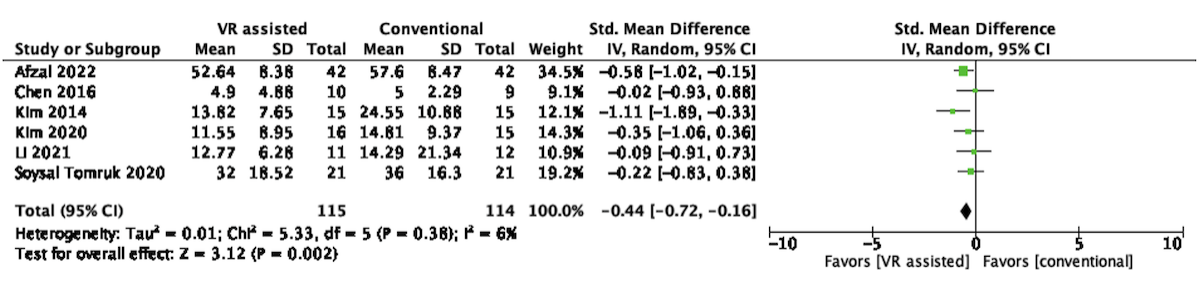
Short-term effects of nonimmersive VR–assisted vs conventional active training in back functional disability. VR: virtual reality.

Of the 28 RCTs, 6 (21.4%; n=229, 20%, participants) [45,48,54,55,58,67] were eligible for pooling. The ODI was extracted over the RMDQ in the pooling because of its higher reliability and relatively lower measurement error [71]. Results favored the use of nonimmersive VR over conventional active training in improving back disability in the short term (SMD –0.44, 95% CI –0.72 to –0.16; *P*=.002) and of low heterogeneity (*I*^2^=6%), as shown in Figure 4.

#### Kinesiophobia

**Figure 5:**
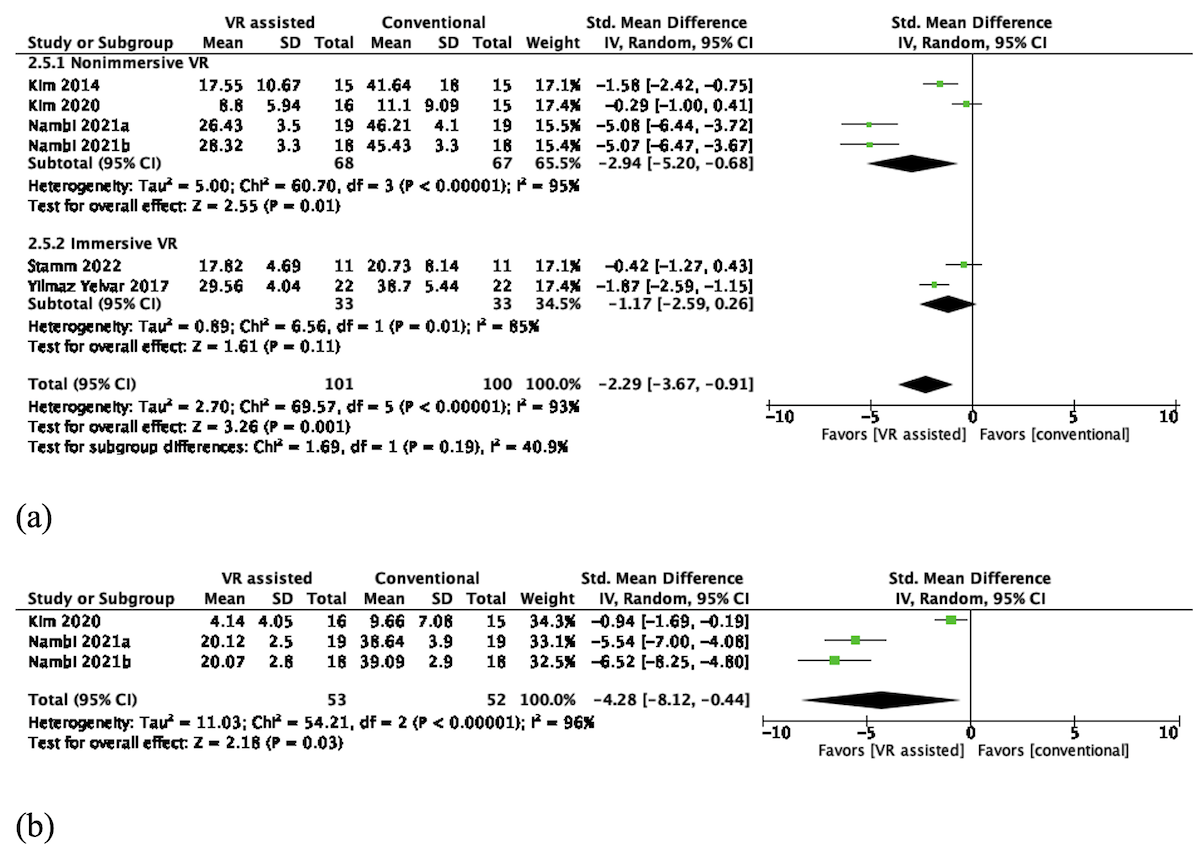
(a) Short-term effects of VR-assisted vs conventional active training in back kinesiophobia (nonimmersive VR via flat screen; immersive VR via HMDs). (b) Intermediate effects of nonimmersive VR–assisted vs conventional active training in back kinesiophobia. HMD: head-mounted device; VR: virtual reality.

In the short term, pooled results of 5 (17.9%; n=135, 11.8%, participants) of the 28 RCTs [45-47,55] favored nonimmersive VR over conventional active training (SMD –2.94, 95% CI –5.20 to –0.68; *P*=.01), with high heterogeneity (*I*^2^=95%). Pooled results of immersive VR from 2 (7.1%; n=66, 5.8%, participants) RCTs were statistically insignificant (SMD –1.17, 95% CI –2.59 to 0.26; *P*=.11), with high heterogeneity (*I*^2^=85%), as shown in Figure 5a.

In the intermediate term, 3 (10.7%; n=105, 9.2%, participants) RCTs [45-47] were pooled. Pooled results favored nonimmersive VR in kinesiophobia (SMD –4.28, 95% CI –8.12 to –0.44; *P*=.03), with high heterogeneity (*I*^2^=96%), as shown in Figure 5b.

### Pain Region: Neck

#### Pain Intensity

**Figure 6:**
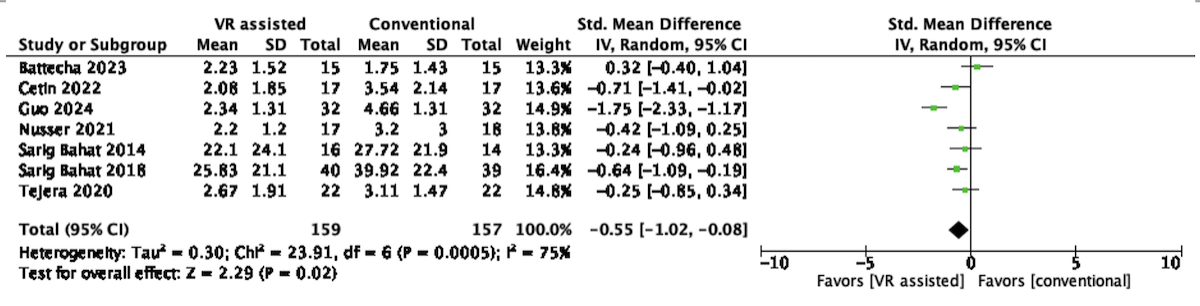
Short-term effects of immersive VR–assisted vs conventional active training in neck pain intensity. VR: virtual reality.

Of the 28 RCTs, 7 (25%; n=316, 6.9%, participants) [49-53,65,69] were eligible for pooling for immersive VR. Pooled results favored immersive VR over conventional active training in reducing pain intensity in the short term (SMD –0.55, 95% CI –1.02 to –0.08; *P*=.02), with high heterogeneity (*I*^2^=75%), as shown in Figure 6.

#### Functional Disability

**Figure 7:**
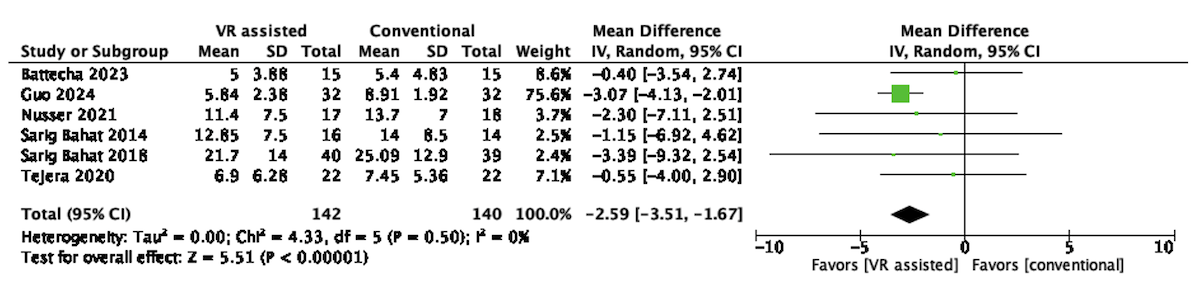
Short-term effects of immersive VR–assisted vs conventional active training in neck functional disability. VR: virtual reality.

Of the 28 RCTs, 6 (21.4%; n=282, 24.7%, participants) [49-52,65,69] were eligible for pooling for immersive VR. Pooled results favored immersive VR over conventional active training in reducing neck disability in the short term (MD –2.59, 95% CI –3.51 to –1.67; *P*<.001) and no heterogeneity (*I*^2^=0%), as shown in Figure 7.

#### Kinesiophobia

**Figure 8:**
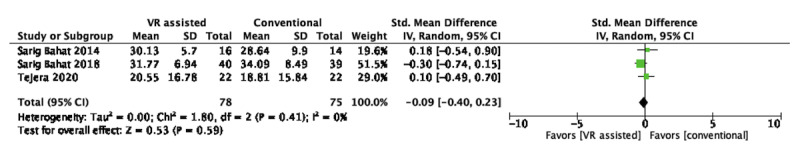
Short-term effects of immersive VR–assisted vs conventional active training in neck kinesiophobia. VR: virtual reality.

Of the 28 RCTs, 3 (10.7%; n=153, 13.4%, participants) [49-51] were eligible for short-term pooling. Pooled results showed no significant effect of immersive VR no kinesiophobia (SMD –0.09, 95% CI –0.40 to 0.23; *P*=.59) and no heterogeneity (*I*^2^=0%), as shown in Figure 8.

### Pain Region: Knee

#### Pain Intensity

**Figure 9:**
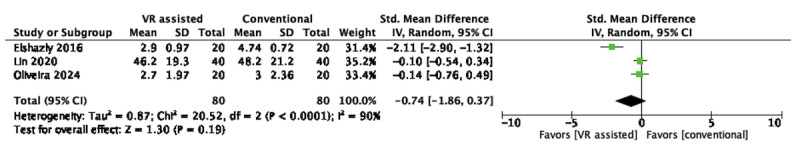
Short-term effects of nonimmersive VR–assisted vs conventional active training in knee pain intensity. VR: virtual reality.

Of the 28 RCTs, 3 (10.7%; n=160, 14%, participants) [42,61,68] on nonimmersive VR were pooled in the short term (SMD –0.74, 95% CI –1.86 to 0.37; *P*=.19), with high heterogeneity (*I*^2^=90%), as shown in Figure 9.

#### Functional Disability

**Figure 10:**
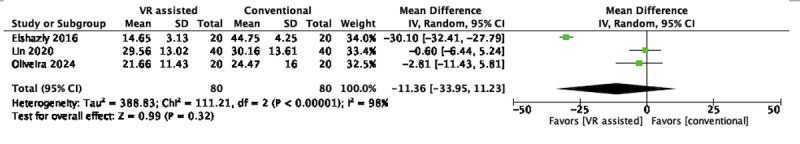
Short-term effects of nonimmersive VR–assisted vs conventional active training in knee functional disability. VR: virtual reality.

Of the 28 RCTs, 3 (10.7%; n=160, 14%, participants) [42,61,68] were pooled in the short term. Pooled results (MD –11.36, 95% CI –33.95 to 11.23; *P*=.32) suggested no statistical and clinical significance for nonimmersive VR versus controls, with high heterogeneity (*I*^2^=98%), as shown in Figure 10.

However, due to the small number of studies, pooling was not possible for chronic shoulder, hip, and other joint pain subgroups.

### Quality of Evidence With the GRADE Approach

The overall quality of evidence ranged from very low to moderate in both nonimmersive VR (Table 1) and immersive VR (Table 2). In low back pain, the assessment showed very low certainty for both nonimmersive and immersive VR–assisted physical therapy in reducing pain intensity and kinesiophobia in the short and the intermediate term and low certainty for nonimmersive VR in reducing functional disability. For neck pain, we found low-to-moderate certainty for immersive VR–assisted physical therapy in reducing pain, functional disability, and kinesiophobia. For knee pain, very low certainty for nonimmersive VR–assisted physical therapy had no statistical significance for improving pain and functional disability in the short term.

**Table 1:** Summary of findings: nonimmersive VR^a^–assisted training compared with conventional active training.

Pain region and outcomes			Time point^b^		Illustrative comparative risks		SMD^c^/MD^d^ (95% CI), *P* value		Participants (N=1144), n (%)		Studies (N=28), n (%)		Certainty of evidence^e^ (GRADE^f^)
**Back**													
	Pain intensity	Short term		The nonimmersive VR group showed more improvement in pain intensity compared to the control group.		–1.79 (–2.72 to –0.87), *P*<.01		476 (41.6)		13 (46.4)		Very low^g^	
	Pain intensity	Intermediate term		The nonimmersive VR group showed more improvement in pain intensity compared to the control group.		–8.15 (–15.29 to –1.01), *P*<.01		135 (11.8)		4 (14.3)		Very low^g^	
	Functional disability	Short term		The nonimmersive VR group showed more improvement in disability compared to the control group.		–0.44 (–0.72 to –0.16), *P*=.002		229 (20.0)		6 (21.4)		Low^h^	
	Kinesiophobia	Short term		The nonimmersive VR group showed more improvement in kinesiophobia compared to the control group.		–2.94 (–5.20 to –0.68), *P*=.01		135 (11.8)		4 (14.3)		Very low^g^	
	Kinesiophobia	Intermediate term		The nonimmersive VR group showed more improvement in kinesiophobia compared to the control group.		–4.28 (–8.12 to –0.44), *P*=.03		105 (9.2)		3 (10.7)		Very low^g^	
**Knee**													
	Pain intensity	Short term		There was no statistically significant difference between groups.		–0.74 (–1.86 to 0.37), *P*=.19		160 (14.0)		3 (10.7)		Very low^g^	
	Functional disability	Short term		There was no statistically significant difference between groups.		–11.36 (–33.95 to 11.23), *P*=.32		160 (14.0)		3 (10.7)		Very low^g^	

^a^VR: virtual reality.

^b^Time points: “short term” defined as postintervention; “intermediate term” defined as 6 months.

^c^SMD: standardized mean difference.

^d^MD: mean difference.

^e^Certainty of evidence: high, further research is very unlikely to change our confidence in the estimate of effects; moderate, further research is likely to have an important impact on our confidence in the estimate of effects and may change the estimate; low, further research is very likely to have an important impact on our confidence in the estimate of effects and is likely to change the estimate; very low, any estimate of effect is very uncertain.

^f^GRADE: Grading of Recommendations, Assessment, Development, and Evaluations.

^g^Downgraded by 3 levels as the risk of bias was unclear or high in most included studies (–1), there was inconsistency in results (I^2^>50%; –1), there was imprecision due to a large effect size (SMD>0.8 or MD>MCID, 95% CI), but the total sample size was small (–1).

^h^Downgraded by 2 levels as the risk of bias was unclear or high in most included studies (–1), and there was indirectness in comparators (–1).

**Table 2:** Summary of findings: immersive VR^a^–assisted training compared with conventional active training.

Pain region and outcomes in the short term^b^			Results of meta-analysis		SMD^c^/MD^d^ (95% CI), *P* value		Participants (N=1144), n (%)	Studies (N=25), n (%)		Certainty of evidence^e^ (GRADE^f^)
**Back**										
	Pain intensity	There was no statistically significant difference between groups.		0.04 (–1.10 to 1.19), *P*=.94		66 (5.8)		2 (8.0)	Very low^g^	
	Kinesiophobia	There was no statistically significant difference between groups.		–1.17 (–2.59 to 0.26), *P*=.11		66 (5.8)		2 (8.0)	Very low^g^	
**Neck**										
	Pain intensity	Immersive VR reduced pain more compared to the control group.		–0.55 (–1.02 to –0.08), *P*=.02		316 (27.6)		7 (28.0)	Low^h^	
	Functional disability	There was no statistically significant difference between groups.		–2.59 (–3.51 to –1.67), *P*<.001		282 (24.7)		6 (24.0)	Low^i^	
	Kinesiophobia	There was no statistically significant difference between groups.		–0.09 (–0.40 to 0.23), *P*=.59		153 (13.4)		3 (12.0)	Moderate^j^	

^a^VR: virtual reality.

^b^“Short term” was defined as postintervention.

^c^SMD: standardized mean difference.

^c^SMD: standardized mean difference.

^d^MD: mean difference.

^e^Certainty of evidence: high, further research is very unlikely to change our confidence in the estimate of effects; moderate, further research is likely to have an important impact on our confidence in the estimate of effects and may change the estimate; low, further research is very likely to have an important impact on our confidence in the estimate of effects and is likely to change the estimate; very low, any estimate of effect is very uncertain.

^f^GRADE: Grading of Recommendations, Assessment, Development, and Evaluations.

^g^Downgraded by 3 levels as the risk of bias was unclear or high in most included studies (–1), there was inconsistency in results (I^2^>50%; –1), and there was indirectness in comparators (–1).

^h^Downgraded by 2 levels as the risk of bias was unclear in most included studies (–1) and there was indirectness in comparators (–1).

^i^Downgraded by 2 levels as the risk of bias was unclear in most included studies (–1), there was imprecision due to a large effect size (SMD>0.8 or MD>MCID, 95% CI), but the total sample size was small (–1).

^j^Downgraded by 1 level as the risk of bias was unclear or high in most included studies (–1).

## Discussion

### Principal Findings

For back pain, very-low-to-low-certainty evidence suggests that nonimmersive VR–assisted training is superior to conventional training in reducing pain, improving disability, and improving kinesiophobia in the short term, and the superior effects on pain and kinesiophobia are sustained in the intermediate term. The effect sizes detected in this study were large for pain intensity in the short and the intermediate term (ie, –1.50 and –8.15, respectively). The effect size for disability of –0.44 was moderate. The effect sizes were also large for kinesiophobia, at –2.29 in the short term and –4.28 in the intermediate term. For neck pain, low-to-moderate-certainty evidence suggests that immersive VR is effective in reducing pain and disability in the short term; the effect size of –0.55 was moderate. The mean difference in disability was –2.59, which was lower than the MCID for a neck disability change of –7.5 [72]. However, no statistically significant effects were detected on kinesiophobia in both short and intermediate terms. For knee pain, only nonimmersive VR was available, and we did not detect any statistically significant difference between nonimmersive VR and control groups in knee pain and function. There are only a few studies on other pain regions, such as the shoulder, hip, and mixed musculoskeletal regions, so pooling was not possible, and the evidence was inconclusive. Our findings suggest that VR-assisted training is superior to conventional active training in managing chronic musculoskeletal pain.

When investigating the effect size of the included studies, especially in nonimmersive VR pooling for chronic back pain, we found that 5 main studies [44,46,47,63,67], which were supervised by physiotherapists in both intervention and control arms, showed large short- and intermediate-term pain reduction effects. Supervision in exercise therapy enhances treatment adherence, achieving the high dosage necessary to demonstrate the VR-assisted effect [73]. Conversely, 5 studies [50,52,53,65,69] on immersive VR–assisted training for neck pain incorporated conservative treatments, such as strengthening and kinematic exercises, to augment their therapeutic effects. These differences in intervention and control designs may have introduced heterogeneity across trials, alongside variations in the participants’ mean age, the athletes’ training background, the dosage of interventions, and the diversity of different VR hardware and software. For example, the hardware included horse simulators, the ProKin system, the Nintendo Wii system, Kinect Xbox 360, the Biodex Balance system, and high-definition television equipped with motion sensors. Although heterogeneity was high, it was inevitable in VR trials due to the unique features of innovations in digital technology. Therefore, we suggested that the high heterogeneity might affect the generalizability of results but should not demerit the clinical effects of VR in reducing back and neck pain.

Comparing the clinical effectiveness of immersive and nonimmersive VR is challenging because most studies on chronic low back pain have used nonimmersive VR, while those on chronic neck pain have used immersive VR. This discrepancy may be due to the use of HMDs in detecting cervical kinematics and range of motion during active training for neck pain, which is not required in studies on back pain. Our systematic review revealed that most studies on back pain have used software comprising ready-made recreational VR games or virtual simulated environments. Meanwhile, immersive therapeutic software aimed at creating presence, learning, and habit building has recently emerged for treating chronic low back pain [74]. This development extends the usefulness of immersive VR–assisted interventions by improving pain interference with activity, mood, and stress, which are commonly found in patients with chronic musculoskeletal pain.

### Comparison With Other Reviews

Ahern et al [19] conducted a review of VR in patients with neck and back pain, with only 2 trials eligible for quantitative synthesis; they nevertheless reached the same conclusion that VR is effective in reducing back and neck pain intensity. Brea-Gómez et al [75] conducted a comprehensive review of studies on chronic back pain; 14 studies were included in the systematic review and 11 in the meta-analysis. Similar to our results, significant differences were found in favor of VR compared to control interventions in pain intensity and kinesiophobia in the short term, with effect sizes of –1.92 and –8.96, respectively; although they showed a trend favoring VR in reducing disability, only 2 trials were included in pooling, and the results were not statistically significant [75]. We included 6 trials in our pooling, allowing us to detect a more accurate effect size, with smaller CIs and statistical significance. Bordeleau et al [76] also reviewed the use of VR in back pain; 16 trials were included in the meta-analysis, and similar results were found, with VR statistically significantly improving back pain intensity over control interventions. Yet, the authors did not evaluate the role of VR in back pain disability and did not analyze the effects based on the level of immersiveness [76]. Li et al [77] found similar immediate VR effects on back pain but not at 3-6 months, possibly due to high heterogeneity and inconsistency from pooling waitlist controls, violating the assumption of a common effect size [78]. Our findings on reducing pain intensity and disability were also similar to those of the systematic reviews conducted by Guo et al [79] and Brea-Gomez et al [80]. Byra and Czernicki [81] reviewed the effectiveness of VR rehabilitation in knee and hip osteoarthritis with or without arthroplasty; meta-analysis was not performed due to heterogeneous study populations and outcome measurements [81]. Although we found a trend favoring the use of VR in knee pain, the small number of studies limited its power to detect statistical significance [81]. Kantha et al [82] supported the use of VR over conventional physical therapy in reducing pain but not in improving disability; their meta-analysis of 5 included studies also favored the use of nonimmersive VR, which was similar to our results [82]. Yet, their results were drawn from pooling of mixed pain regions.

### Strengthens and Limitations

The strengths of this study include a comprehensive review of VR in different musculoskeletal pain regions, not only in the short term but also in the intermediate term. This is the first study to evaluate the degree of immersiveness in VR-assisted active training on validated pain outcomes. We used a rigorous methodology that conformed to best-practice guidelines [16].

There were several limitations. Although we increased the number of VR studies, the total participant sample size was still small, and quantitative syntheses included a small number of studies in most comparisons. For the same reason, we were unable to generate funnel plots to assess publication bias for most outcomes [35]. Finally, since chronic pain is a biopsychosocial condition, the inclusion of only VR-assisted active training and the exclusion of VR-assisted psychotherapy might potentially underestimate the true effect of VR on chronic pain [83].

### Future Research and Clinical Implications

Future research needs to focus on the long-term effects of VR-assisted active training on chronic pain management. The joy and pleasure associated with VR interactions are attractive, but the excitement will fade with time. Therefore, it is essential to evaluate participants’ adherence to VR interventions [9,84]. Furthermore, trials should be conducted to evaluate other pain, especially knee pain, given that it is the most prevalent condition in the aging population [85]. The mechanism of action of VR in pain regulation will need to be elucidated for the best design of VR apps. Finally, cost-effectiveness should be evaluated to inform resource allocation of VR in clinical practice.

### Conclusion

In summary, our study found that nonimmersive VR–assisted active training is superior to conventional active training in reducing pain intensity, functional disability, and kinesiophobia in low back pain in the short and the intermediate term. Immersive VR–assisted active training is effective in reducing the intensity of neck pain. Evidence on knee pain, shoulder pain, and hip pain remains inconclusive due to the small number of studies. Further high-quality VR trials with longer-term follow-up, adequate sample sizes, and cost-effectiveness analysis will inform the role of VR with different immersive levels in chronic musculoskeletal pain management.

## Appendix

Multimedia Appendix 1 Search strategies.

Multimedia Appendix 2 Summary of included studies.

Multimedia Appendix 3 PRISMA checklist.

## Abbreviations

GRADE: Grading of Recommendation Assessment, Development, and Evaluation
HMD: head-mounted device
MCID: minimally clinically important difference
MD: mean difference
ODI: Oswestry Disability Index
PRISMA: Preferred Reporting Items for Systematic Reviews and Meta-Analyses
RCT: randomized controlled trial
RMDQ: Roland-Morris Disability Questionnaire
RoB 2: Cochrane Risk-of-Bias Tool version 2
SMD: standardized mean difference
VR: virtual reality
