# Supplementary material for: Immersive and Nonimmersive Virtual Reality–Assisted Active Training in Chronic Musculoskeletal Pain: Systematic Review and Meta-Analysis
Source: J Med Internet Res. 2024 Aug 19;26:e48787. doi: 10.2196/48787 (PMC11369537; doi:10.2196/48787)
Supplement: Multimedia Appendix 1 [file jmir_v26i1e48787_app1.docx]

**Appendix 1: Search strategies**

1. **PubMed** (Last search on 9^th^ June 2024)

#1 (chronic) AND ("cervical pain"[tw] OR "neck pain"[tw] OR "shoulder pain"[tw] OR "thoracic pain"[tw] OR "back pain"[tw] OR "low back pain"[tw] OR "arthralgia"[tw] OR "joint pain"[tw] OR "knee pain"[tw] OR "ankle pain"[tw] OR "limb pain"[tw] OR "musculoskeletal pain"[MeSH] OR "Neck pain"[MeSH] OR "Shoulder Pain"[MeSH] OR "Back Pain"[MeSH] OR "Arthralgia"[MeSH] OR "Patellofemoral Pain Syndrome"[MeSH] )

#2 "osteoarthritis"[tw] OR "degenerative joint*[tw] NOT structural[tw]” OR “subacromial impingement syndrome”[tw] OR "Osteoarthritis"[MeSH] OR "Osteoarthritis, Spine"[MeSH] OR "Osteoarthritis, Knee"[MeSH] OR "Osteoarthritis, Hip"[MeSH]

#3 "virtual reality*"[tw] OR "augmented reality*"[tw] OR "mixed reality*"[tw] OR "Virtual Reality"[MeSH] OR "Virtual Reality Exposure Therapy"[MeSH] OR "Augmented Reality"[MeSH]

#4 #1 OR #2

#5 #3 AND #4

1. **Scopus** (Last search on 9^th^ June 2024)

( "musculoskeletal pain" OR "cervical pain" OR "neck pain" OR "shoulder pain" OR "thoracic pain" OR "back pain" OR "arthralgia" OR "joint pain" OR "knee pain" OR "ankle pain" OR "limb pain" ) AND "chronic" OR "osteoarthritis AND NOT structural" OR "degenerative joint" AND ( "virtual reality" OR "augmented reality" OR "mixed reality" )

1. **Web of science (**Last search on 9^th^ June 2024)

ALL=(chronic) AND (ALL=(musculoskeletal pain) OR ALL=(cervical pain) OR ALL=(neck pain) OR ALL=(shoulder pain) OR ALL=(thoracic pain) OR ALL=(back pain) OR ALL=(low back pain) OR ALL=(arthralgia) OR ALL=(joint pain) OR ALL=(knee pain) OR ALL=(ankle pain) OR ALL=(limb pain)) AND ALL=(“virtual reality*”) NOT ALL=(systematic review) NOT ALL=(scoping review) NOT ALL=(protocol)
