# Supplementary material for: Immersive and Nonimmersive Virtual Reality–Assisted Active Training in Chronic Musculoskeletal Pain: Systematic Review and Meta-Analysis
Source: J Med Internet Res. 2024 Aug 19;26:e48787. doi: 10.2196/48787 (PMC11369537; doi:10.2196/48787)
Supplement: Multimedia Appendix 2 [file jmir_v26i1e48787_app2.docx]

**Table S1.** Summary of included studies (N=28).

| Author | Pain region(s) | Sample size (N); age (years), mean (SD)/median (IQR); females, (%) | Intervention; sample size, n (%) | Comparator; sample size, n (%) | Outcomes | Frequency and duration | Assessment time points (weeks) | Overall risk-of-bias judgment |
| --- | --- | --- | --- | --- | --- | --- | --- | --- |
| Ditchburn et al [60]^a^ | Joint pain (hips, hands, back) | N=54  Age, mean (SD):  VR^b^, 71.78 (6.1); control, 69.78 (4.48)  Female %:  VR, 81.5; control, 74.1 | Nonimmersive VR: 5 games from the IREX system consisting of a computer installed with VR software, a digital camera, a green fabric screen, and red gloves for 40 minutes (n=27, 50%) | Supervised traditional gym-based exercise for 40 minutes (n=27, 50%) | NRS^c^ (within 30 days, time of test), MAPS^d^ center of pressure, UTAUT^e^ questionnaire, FSS^f^, Borg rating of perceived exertion, Subjective Mental Effort Questionnaire, heart rate | 2 sessions/week for 6 weeks | 0, 6 | Some concerns |
| Pekyavas et al [59]^a^ | Shoulder | N=30  Age, mean (SD): 40.6 (11.7)  Female %: VR, 93.3; control, 86.7 | Nonimmersive VR: supervised interactive sports game (Wii sports, eg, boxing, bowling, tennis) via Nintendo Wii and television (TV) screen for 45 minutes/day (n=15, 50%) | Home exercise program for 45 minutes/day (n=15, 50%) | VAS^g^ (at rest, during activity, at night), SPADI^h^ score, Neer and Hawkin test, scapular retraction and assistance tests, lateral scapular slide test | 2 sessions/week for 6 weeks | 0, 6, 7 | Some concerns |
| Afzal et al [62] | Back | N=84  Mean age: VR, 38.2 (11.8); control: 37.5 (12.5)  Females: VR, 69.04; control: 64.28 | Nonimmersive VR: 10-minute VR exergame with kinetic device Mode V.2 with a motion-sensing input device incorporated with a red-green-blue camera, a time-of-flight sensor, and a liquid crystal display (LCD) screen (n=42, 50%) | Routine physical therapy with 10-minute heat therapy, hamstring stretching, back strengthening, and diagonal elevation of the arm and leg; total therapy duration not reported (n=42, 50%) | NRS, ODI^i^ | 3 sessions/week for 4 weeks | 0, 4, 8, 12 | Some concerns |
| Chen et al [58] | Back | N=19  Age range: 19-30  Female %: not reported | Nonimmersive VR: horse riding simulator (Hongjin Leports) for 15 minutes + control group exercise for 15 minutes (n=10, 52.6%) | Lumbar strengthening exercise for 30 minutes (n=9, 47.4%) | VAS, ODI, LoS^j^ | 3 sessions/week for 4 weeks | 0, 4 | Some concerns |
| Kim et al [55] | Back | N=30  Mean age: VR, 44.33; control, 50.46  Female %: 100 | Nonimmersive VR: yoga program using Wii Fit, 30 minutes per session. (n=15, 50%) | 30-minute trunk stabilization and 30-minute physical therapy (n=15, 50%) | VAS, ODI, RMDQ^k^, FABQ^l^, algometer (kg/cm^2^) | 12 sessions over 4 weeks | 0, 4 | Some concerns |
| Kim et al [45] | Back | N=30  Age, mean (SD): VR, 26 (3.82); control, 28.79 (9.05)  Female %: VR, 31.8; control, 57.7 | Nonimmersive VR: simulated horseback system developed with a horse simulator and screen display for 46 minutes (n=15, 50%) | Stabilization exercise with suspension for 46 minutes (n=15, 50%) | NRS, ODI, RMDQ, FABQ | 16 sessions for more than 8 weeks | 0, 4, 8, 24 | High risk |
| Li et al [54] | Back | N=34  Age, mean (SD): VR + magnetic, 21.91 (2.43); MCE^m^ + magnetic, 23.75 (3.09); control, 23.75 (4.09)  Female %: 73.5 | Nonimmersive VR: “Fruit Ninja” game for 30 minutes/session displayed by Kinect Xbox 360 via a TV screen with magnetic thermal therapy for 20 minutes (n=11, 32.4%) | 30-minute MCE + 20-minute magnetic thermal therapy (n=12, 35.2%)^n^; control, 20-minute magnetic thermal therapy (n=11, 32.4%) | VAS, ODI, mean muscle activation time (transverse abdominis, multifidus, lateral gastrocnemius, tibialis anterior), integrals of electromyography activities | 5 sessions/week for 2 weeks | 0, 2 | Some concerns |
| Matheve et al [43] | Back | N=84  Age, mean (SD): VR, 42.1 (11.5); control, 44.2 (11.9)  Female %: 64 | Nonimmersive VR: 2 VR games (2 minutes each) displayed on a high-definition TV screen, controlled by pelvic tilts with a motion sensor (n=42, 50%); tme of intervention not reported | Pelvic tilts according to a beep tone. (n=42, 50%); time of intervention not reported | Pain intensity | A single session | 0 (baseline, during, and after exercise) | Some concerns |
| Monteiro-Junior et al [57] | Back | N=30  Age, mean (SD): 68 (4)  Female %: 100 | Nonimmersive VR: Nintendo Wii-motion and Wii Balance Board for 30 minutes + control exercise for 60 minutes (n=16, 53.3%) | Core and strength training for 60 minutes (n=14, 46.7%) | NRS, balance measures from Wii Balance Board, sit-to-stand test, profile of mood states | 3 sessions/week for 8 weeks | 0, 8 | Some concerns |
| Nambi et al [44] | Back | N=45  Age, mean (SD): VR, 21.25 (1.2); IKT^o^, 20.23 (1.6); conventional, 20.78 (1.6)  Female %: 0 | Nonimmersive VR: game of ball shooting with the ProKin system PK 252N Techno body for 30 minutes + physiotherapy^p^ for 25 minutes (n=15, 33.3%) | IKT with isokinetic dynamometer + physiotherapy for 25 minutes (n=15, 33.3%), conventional balance training + physiotherapy for 25 minutes (n=15, 33.3%)^n^ | VAS, player wellness, sports performance analysis (40 m sprint, 4×5 m sprint), submaximum shuttle run, jump performance (countermovement jump, squat jump) | 5 sessions/week for 4 weeks | 0, 4, 8, 24 | Some concerns |
| Nambi et al [46] | Back | N=58  Age, mean (SD): VR, 23.2 (1.5); IKT, 22.8 (1.6); conventional, 23.3 (1.5)  Female %: 0 | Nonimmersive VR: game focusing on balance of stability via the ProKin system PK 252N for 30 minutes + 25-minute physiotherapy^p^ (n=19, 32.8%) | IKT with isokinetic dynamometer + 25-minute physiotherapy (n=20, 34.5%); conventional balance training for core muscles of the trunk + 25-minute physiotherapy (n=19, 32.8%)^n^ | VAS, TSK^q^, blood serum analysis (glucose, insulin, HOMA-IR^r^, growth hormone, prolactin, ACTH^s^, cortisol) | 5 sessions/week for 4 weeks | 0, 4, 24 | Some concerns |
| Nambi et al [47] | Back | N=54  Age, mean (SD): VR, 22.3 (1.6); combined physical rehabilitation, 21.4 (1.8); conventional balance training, 21.9 (1.8)  Female %: 0 | Nonimmersive VR: shooting game via the ProKin system PK 252N for 30 minutes + 25-minute physiotherapy^p^ (n=18, 33.3%) | Balance training for core muscles through a Swiss ball + 25-minute physiotherapy. (n=18, 33.3%); conventional balance training for core muscles + 25-minute physiotherapy (n=18, 33.3%)^n^ | VAS, TSK, blood serum analysis (glucose, insulin, HOMA-IR, growth hormone, prolactin, ACTH, cortisol) | 5 sessions/week for 4 weeks | 0, 4, 24 | Some concerns |
| Nambi et al [63] | Back | N=58  Age, mean (SD): VR, 23.2 (1.6); IKE, 22.9 (1.7); conventional, 22.8 (1.8)  Female %: 0 | Nonimmersive VR: car racing game conducted via ProKin, Techno body, Italy + strength training focused on trunk muscles for 30 minutes (n=19, 32.8%) | Isokinetic exercise training in isokinetic device (Humac Norm, Stoughton); total treatment duration not reported (n=19, 32.8%); conventional routine balance exercise for 10-15 repetitions/day and 3 repetitions of 15 seconds for lower limb muscles (n=20, 34.5%)^n^ | VAS, maxVO2, heart rate, cross-sectional MRI^t^, muscle thickness, inflammatory biomarkers | 5 sessions/week for 4 weeks | 0, 4 | Some concerns |
| Park et al [56] | Back | N=24  Age, mean (SD): VR, 44.12 (5.48); LSE^u^, 43.37 (5.42); control, 45.50 (5.34)  Female %: 0 | Nonimmersive VR: Nintendo Wii sports program (including wakeboard, Frisbee dog, jet ski, and canoe games) with TV screens and motion sensors for 30 minutes + control physical therapy for 50 minutes (n=8, 33.3%) | LSE for 30 minutes + control physical therapy for 50 minutes (n=8, 33.3%)^n^; control physical therapy (hot pack for 30 minutes + interferential current therapy for 15 minutes + deep heat with ultrasound for 5 minutes (n=8, 33.3%) | VAS, isometric lifting strength for back strength, one-legged stand test, RAND-36^v^ | 3 sessions/week for 8 weeks | 0, 8 | Some concerns |
| Soysal Tomruk et al [67] | Back | N=42  Age, median (IQR): VR, 46 (40.05-50.50); control, 45 (44-48)  Female %: not reported | Nonimmersive VR: computer-based stability training via the Biodex Balance System for 30 minutes (n=21, 50%) | Postural control exercise supervised by physiotherapist for 30 minutes (n=21, 50%) | NRS, ODI, LoS, total energy expenditure | 2 sessions/week for 12 weeks | 0, 12 | Some concerns |
| Stamm et al [64] | Back | N=22  Age, mean (SD): 75 (5.8)  Female %: VR, 72.7; control, 54.4 | Immersive VR: supervised physiotherapeutic intervention with a VR HMD^w^ headset using the ViRST VR app consisting of 12 exercises that includes warm up, abdominal and back strengthening, core stability, stretching, and educative units for 30 minutes (n=11, 50%) | Supervised conventional sitting gymnastics consisting of 12 exercises identical to those in the VR group for 30 minutes (n=11, 50%) | NRS, Chronic Pain Grade Questionnaire, Hannover Functional Ability Questionnaire (Ffb-H-R), TSK-11, health survey 12-item Short Form Survey (SF-12), technology usage inventory (TUI), User Experience Questionnaire (UEQ) | 3 sessions/week for 4 weeks | 0, 4 | Some concerns |
| Yilmaz Yelvar et al [48] | Back | N=44  Age, mean (SD): VR, 46.27 (10.93); traditional walking exercise, 52.81 (11.53)  Female %: VR, 45.5; traditional walking, 81.8 | Immersive VR: virtual walking video clip played with video glasses (Wrap920) + physical therapy same as the control group (n=22, 50%); VR intervention time not reported | Physical therapy^x^ (n=22, 50%) | VAS, TSK, ODI, timed-up-go test, 6-minute walk test, Nottingham Health Profile | 5 sessions/week for 2 weeks | 0, 2 | Some concerns |
| Battecha et al [65] | Neck | N=30  Age, mean (SD): VR, 21.23 (0.83); control, 21.26 (0.79)  Female %: 100 | Immersive VR: BOBOVR Z-6 model attached to Samsung Galaxy A30s smartphone with “VR Ocean Aquarium” (HY Games Version 1.0.25) + 10 repetitions of strengthening exercise (n=15, 50%); VR intervention time not reported | Supervised stretching exercises and isometric strengthening for 10 minutes (n=15, 50%) | VAS, NDI^y^, range of motion, pressure pain threshold | 3 sessions/week for 6 weeks | 0, 6 | High |
| Cetin et al [53] | Neck | N=34  Age, mean (SD): VR, 40.00 (11.88); control, 41.94 (10.76)  Female %: VR, 70.5; control, 64.7 | Immersive VR: 20-minute neck movement with Oculus Go VR HMD with Ocean Rift and Gala 360 VR apps installed + MCE for 20 minutes (n=17, 50%) | MCE including strengthening of deep cervical flexors, deep cervical extensors, and axioscapular muscles; stretching exercises; and postural correction exercises for 40 minutes (n=17, 50%) | VAS, range of motion and joint position sense error (JPSE), pain pressure threshold (PPTs), Profile Fitness Mapping Neck Questionnaire (ProFitMap-Neck), HADS^z^, 36-item Short-Form Survey (SF-36) | 3 sessions/week for 6 weeks | 0, 6 | Some concerns |
| Guo et al [69] | Neck | N=64  Age, mean (SD): VR, 35.94 (11.02); control, 40.09 (11.97)  Female %: VR, 78; control: 69 | Immersive VR: Pico G2 4k head-mounted VR glasses, monitor screen, and optical motion capture camera, 20-minute game that included 3 modules (range of motion, proprioception, and velocity) + 5-minute active exercise and 5-minute transcutaneous electrical nerve stimulation (n=32, 50%) | 30-minute Conventional rehabilitation: 15-minute active exercise modalities + 15-minute transcutaneous electrical nerve stimulation therapy (n=32, 50%) | NRS, NDI, cervical range of motion, proprioception, mean and peak velocity, global perceived effect, patient satisfaction and relief of symptoms | 10 sessions over 4 weeks | 0 (baseline and immediately postintervention), 12 | Some concerns |
| Nusser et al [52] | Neck | N=51  Age, mean (SD): VR, 51.2 (8.8); sensorimotor, 53.1 (5.7); control, 49.8 (8.1)  Female %: VR, 53; sensorimotor, 69; control, 66 | Immersive VR: 20-minute neck-specific sensorimotor training while wearing a helmet (Schutzhelm uvex pheos alpine) with an integrated monitor (VR 5DT HMD 8000-26 2D), 3Space Fastrak System used for head movement tracking + control group rehabilitation program (n=17, 33.3%) | Sensorimotor group: 20-minute general sensorimotor training + control group rehabilitation program (n=16, 31.4%)  Control group: supervised rehabilitation program that included individual and group therapies instructed by physiotherapists and certified sports scientists; treatment duration not reported. (n=18, 35.3%)^n^ | NRS (neck pain at rest^n^, neck pain during motion, headache at rest, headache during motion), sctive cervical range of motion, NDI | 6 sessions over 3 weeks | 0, 3 | Some concerns |
| Rezaei et al [66]^a^ | Neck | N=42  Age, mean (SD): VR, 36.19 (9.8); control, 31.23 (9.49)  Female %: VR, 42.9; control, 52.4 | Nonimmersive VR: video game that lasted for 21 minutes (Cervigame version 1.01), displayed on a monitor screen; Head Mouse Extreme (Origin Instruments Corporation) used to detect head movements (n=21, 50%) | Conventional proprioceptive training for 21 minutes, consisted of eye follow, gaze stability, eye-head coordination, and position sense and movement sense practice (n=21, 50%) | VAS, NDI, Y-balance test | 8 sessions over 4 weeks | 0, 5 | Some concerns |
| Sarig Bahat et al [50] | Neck | N=26  Age, mean (SD): VR, 40.63 (14.18); kinematic, 31.13 (12.59)  Female %: VR, 64.7; kinematic, 68.8 | Immersive VR: HMD with a 3D motion tracker built in (Wrap 1200VR by Vuzix) to control a virtual pilot flying a red airplane for 15-20 minutes + kinematic training for 10-15 minutes + 30-minute home exercise (n=14, 53.8%) | Kinematic exercise including a head pursuit task conducted with a laser pointer and a poster for 30 minutes + 30-minute home exercise (n=12, 46.2%) | VAS, NDI, TSK, VR kinematics (range of motion, peak velocity, mean velocity, time to peak velocity in percentage, SD of statis head sway, accuracy), sensorimotor (eyes closed balance, single-leg stance, step test) | 3 sessions/week for 12 weeks | 0, 12 | Some concerns |
| Sarig Bahat et al [51]^aa^ | Neck | N=56 in phase 2  Age, median: control group in phase 1, 48; no age median reported for phase 2  Female %: phase 1 control group, 77 | Immersive VR:  VR training group, customized neck VR system (Oculus Rift DK1 HMD equipped with 3D motion tracking) for 20 minutes/session (n=29, 51.8%) | Kinematic training conducted with a head-mounted laser beam aimed at a 70×70 cm poster for 20 minutes (n=27, 48.2%) | VAS, NDI, global perceived effect, EQ-5D, TSK, cervical motion kinematics outcome measures (velocity, time to peak velocity percentage, number of velocity peaks, accuracy error, cervical range of motion) | 4 sessions/week for 4 weeks | 0, 4, 12 | Some concerns |
| Tejera et al [49] | Neck | N=44  Age, mean (SD): 29.7 (10.81)  Female %: 52.3 | Immersive VR:  2 VR games “Fulldive VR” and “VR Ocean Aquarium 3D” delivered via VR Vox Play glasses with an HMD clamping system with a smartphone (LGQ6); total intervention time not reported. (n=22, 50%) | Neck exercises (including flexion, extension, rotation, and lateral flexion); total intervention time not reported. (n=22, 50%) | VAS, conditional pain modulation, temporal summation, active cervical range of movement, NDI, pain catastrophizing, TSK, FABQ, pain pressure thresholds, pain-related anxiety | 2 sessions/week for 4 weeks | 0, 4, 12 | Some concerns |
| Elshazly et al [42] | Knee | N=60  Age, mean (SD): virtual reality therapy + CET^ab^, 58 (6); SMT + CET, 60 (8); CET only, 59 (7)  Female %: not reported | Nonimmersive VR: VR game “Light Race” displayed on the screen for 15-30 minutes, combined with CET (n=20, 33.3%) | Sensorimotor therapy according to 3 phases (duration not reported), combined with CET (n=20, 33.3%); CET, 5-minute warm-up + 12-minute walking + 5-minute cool-down (n=20, 33.3%)^n^ | VAS, position sense score, WOMAC^ac^, health-related quality of life (HRQOL) | 3 sessions/week for 8 weeks | 0, 8 | Some concerns |
| Lin et al [61] | Knee | N=80  Age, mean (SD): VR, 55.9 (15.8); control, 58.1 (16.9)  Female %: 51.25 | Nonimmersive VR: supervised 20-minute active video game treatment using the Hot Plus system installed with 2 games, “Whack-a-Mole” and “Archery,” with 3 different levels, combined with 20-minute hot pack, 20-minute transcutaneous electrical nerve stimulation (n=40, 50%) | Supervised 20-minute therapeutic exercise including stabilization and strength and balance exercises, combined with 20-minute hot pack, 20- minute transcutaneous electrical nerve stimulation (n=40, 50%) | WOMAC, static and dynamic balance, physical functional performance, WHOQOL-BREF, HADS, Multidimensional Fatigue Inventory (MFI), Work Ability Index, Chronic Pain Grade Questionnaire | 3 sessions/week for 4 weeks | 0, 2, 4, 8, 16 | Some concerns |
| Oliveira et al [68] | Knee | N=40  Age, mean (SD): VR, 62.36 (7.39); control, 62.60 (8.62)  Female %: VR, 70; control, 85 | Nonimmersive VR training using Xbox 360 video game with Microsoft Kinect 360 sensor, 20-minute game session + 30-minute kinesiotherapy (n=20, 50%) | 30-minute Kinesiotherapy + 20-minute postural control and balance training (n=20, 50%) | Altered anticipatory postural adjustments (APAs), Mini-Balance Evaluation Systems Test, WOMAC, VAS | 2 sessions/week for 8 weeks | 0 (baseline and postintervention) | Some concerns |

^a^Qualitative synthesis only.

^b^VR: virtual reality.

^c^NRS; Numerical Rating Scale.

^d^MAPS: Multi Affect and Pain Survey.

^e^UTAUT: Unified Theory of Acceptance and Use of Technology.

^f^FSS: Flow State Scale.

^g^VAS: Visual Analog Scale.

^h^SPADI: Shoulder Pain and Disability Index.

^i^ODI: Oswestry Disability Index.

^j^LoS: limit of stability.

^k^RMDQ: Roland-Morris Disability Questionnaire.

^l^FABQ: Fear-Avoidance Beliefs Questionnaire.

^m^MCE: motor control exercise.

^n^Extracted data as comparator in meta-analysis.

^o^IKT: isokinetic therapy.

^p^Physiotherapy was composed of a home-based exercise protocol, 20-minute hot pack therapy, and 5-minute ultrasound.

^q^TSK: TAMPA Scale of Kinesiophobia (17-items).

^r^HOMA-IR: homeostasis model assessment-insulin resistance.

^s^ACTH: adrenocorticotropic hormone.

^t^MRI: magnetic resonance imaging.

^u^LSE: lumbar stabilization exercise.

^v^RAND-36: Rand 36-item Health Status Inventory.

^w^HMD: head-mounted device.

^x^Physical therapy was composed of 15-minute hot-pack therapy, 15-minute transcutaneous electrical nerve stimulation, 5-minute deep heat with ultrasound, and therapeutic exercises (treatment duration was not reported).

^y^NDI: Neck Disability Index.

^z^HADS: Hospital Anxiety and Depression Scale.

^aa^Sarig Bahat et al [51] conducted a randomized controlled trial (RCT) in 2 phases. In phase 1, participants were separated into 3 limbs: VR training, laser training, and waitlisted control group. For phase 2, the waitlisted control participants from phase 1 (n=25) and 7 additional participants were recruited. Only outcomes reported in phase 2 were used in this meta-analysis because the setting of phase 2 correlated more with our research question.

^ab^CET: conventional exercise therapy.

^ac^WOMAC: Western Ontario and McMaster Universities Osteoarthritis Index.
